# Clinical Notes

**Published:** 1884-12

**Authors:** J. T. Kent


### CLINICAL NOTES.

Alex. Kerr, æt. forty-eight, consulted me April 1st with a very painful chronic spasm of the right side of the face. This annoying complaint began five years ago. It was called a neuralgia, and after taking crude drugs for a year or more without benefit the lamented Hodgen resected a portion of the inferior dental nerve, which was followed by relief for six months, when the pain and spasm returned with full violence, giving him no rest during the day. Some two years ago he was again operated on at the Missouri Medical College clinic by cutting down upon the dental foramen, seizing the inferior dental, and drawing it out an inch and cutting it off close up to the foramen. This was followed by relief for several months.

At present, when he desires to sleep, he lies quietly without moving a muscle, and he soon sleeps, but if at any time during the night while moving his body he also moves his jaw, the pain and the spasm return and rend until he is nearly wild with suffering; finally he forces himself to be quiet, and he again sleeps.

Hard pressure relieves; hence while sitting in my office he held his hand pressed hard against the right side of the face. When he was compelled to talk to answer my numerous questions, the first motion would cause a return of the spasm.

While chewing and swallowing he suffered intensely, and he ameliorated the pain by pressing hard against the cheek. All the muscles of the right cheek seemed to take part in this violent spasmodic action. The pain was present only during the spasm. The violent spasm seemed to cause the pain by the violent tension of the nerve filiment within. I could glean no other wrongs or symptoms. He was a very hardy fellow, and said he could do any kind of labor if this jerking would only go away. This patient had always been treated pathologically, and remedies had no effect when selected by this extremely fallible guide. We shall see what our symptomotology can do, and how it does it. It seems strange that a system of therapeutics several thousand years old should fail to cure such a simple little trouble.

The case is so very simple and the cure so very quick, I suspect that a tyro in the "art of healing" could not fail to cure this very simple case.

Chronic spasms of the cheek: *Agar.*, Bell., Bry., Calc. c., Caust, Con., Hep., Dulc., Lach., Lyc., *Nat-m.*, Nux.

Chronic spasms of the cheek, especially the right side: *Bell.*, *Calc-c.*, Caust., Nat-m., *Nux.*

Swallowing and motion aggravated: Bell., Bry., Calc., Con., Hep., Lach., Lyc., Nat-m., Nux., and many others not having the chronic spasms of cheek.

It will be seen that Nux and Nat-m. are to be compared. This may have been of malarial origin, but what help is that, as there was no choice between Nux and Nat-m. on that ground.

There was no special time of aggravation that specially corresponded to Nat-m., but to favor the *choice* of Nux he was of Nux constitution, and he had been drugged for five years.

With all these in view I could not well rest on any remedy but Nux, which was given at once, $2^{m}$ (Jenichen).

The intensity of the spasm was lessened gradually, and in five days it was no more. The single prescription cured. I know what men say who do not follow the law of Hahnemann. But their sin be on their own heads, as the opportunities for learning this art are within the reach of every man.

The inductive method is the only one known to give satisfaction, which method must be followed in every particular or the result cannot be predicted. J. T. Kent.

