# Erratum

**Published:** 1885-01

**Authors:** 


ERRATUM.—In our December ('84) issue, bottom of page 360 and top of page 361, for *chronic* spasms of cheek, read *clonic* spasms, etc.

